# Feasibility Evaluation of Local Laser Treatment for Strengthening of Thin-Walled Structures from Low-Carbon Steel Subjected to Bending

**DOI:** 10.3390/ma13143085

**Published:** 2020-07-10

**Authors:** Oleksandr Kapustynskyi, Nikolaj Višniakov, Darius Zabulionis, Artur Piščalov

**Affiliations:** Faculty of Mechanics, Vilnius Gediminas Technical University, Saulėtekio al. 11, 10221 Vilnius, Lithuania; oleksandr.kapustynskyi@vgtu.lt (O.K.); darius.zabulionis@vgtu.lt (D.Z.); artur.piscalov@vgtu.lt (A.P.)

**Keywords:** reinforcing, laser treatment, strengthening, bending, low-carbon steel, thin steel plate

## Abstract

This paper is devoted to investigating numerically, by finite element analysis (FEA), and analytically the influences and effects of laser processing of the surface of thin-plate, low-carbon structural steel. The plate mechanical properties—axial and flexural stiffnesses, force-deflection behavior and cross-section force-strain behavior—are investigated after different laser treatments. An analytical methodology of the estimation of the cross-section area of the laser-processed metal is also proposed in the present article, that can be applied to choosing the reasonable distance between the centers of the laser-processed tracks. The methodology takes into account the width of the laser-processed tracks and the distances between these tracks. The experimental, finite element numerical and analytical analyses showed that the laser treatments of the surface of the steel plate increase the yield point of the laser-processed metal and the axial and flexural stiffnesses of the plate.

## 1. Introduction

High-quality carbon and low-alloyed steels are widely used to manufacture thin-walled metal products like tubes, vessels, beams, shells, panels, folds, membrane structures and other forms used in metalworking. However, application of these cheap steels has been limited due to inadequate mechanical strength, stiffness and low resistance to corrosion [1]. Reducing the weight of steel structures and the amount of metal required by improving the strength, hardness and stiffness of steel is a major task in the metalworking sector. 

Thin-walled shell structures are lightweight constructions which are mostly designed to carry only tension loads, without taking into account compression or bending. However, such three-dimensional tensile structures can be subjected to unplanned bending loads and bending stresses due to concentrated transversal forces. These forces can be accidental impact, concentrated external or support/reaction forces, which may cause significant damage or failure of the whole construction [2]. 

Different design strategies are useful to avoid failure or the risk of collapse of the metal structure, and to increase the resistance to collapse or sensitivity of the structure to unplanned heavy loads. Heavily loaded thin-walled structural elements, such as the shell structures of buildings, rockets, chemical reactors, thin-walled pipes, vessels and other metal structures, are designed using special calculation methods that take plastic deformation into account. Then, calculations are carried out beyond the elasticity of the metals. The ultimate load method or limit-state design allows metal consumption to be reduced and gives access to additional reserves of structural strength in thin-walled steel structures. It also increases available workloads or ultimate bending moments in comparison with the results achieved by traditional working stress methods in the elastic stage [3,4].

Different heat-treatment or thermochemical treatment processes are very popular finishing techniques in metalworking, and can be useful to increase the yield, tensile strength and hardness of steel structures [5]. 

In practice, only steels with carbon content above 0.3% are reliably heat-treatable to improve their mechanical properties by formation of martensitic structures. Theoretically, steels with carbon content below 0.3% can be strengthened by refining grain size and using a sufficient cooling rate during traditional quenching. The critical cooling rate for low-carbon steels with 0.19 wt.% must be around 1925 °C/s [6], but the rate at which they must be cooled to produce martensite is so high that it cannot be attained by traditional quenching in water or induction hardening.

Furthermore, heat-treatment and thermomechanical treatment techniques are very expensive and complicated. The strengthening of the metal sheets by means of strengthening elements and reinforcing ribs, special steel profiles and sections of complex geometry or local thickenings of ribs, is a well-known engineering solution [2,3]. However, the application of geometric improvements increases the manufacturing costs and the weight of metal parts, and requires complex manufacturing methods, expensive metalworking equipment and tooling, special designs and very experienced engineering staff.

An alternative way to manufacture metal structures with better strength and stiffness can involve methods for fabricating rib-strengthened or grid-strengthened composite structures. All dual-phase steels have heterogeneous microstructures, similar to natural composite materials, the properties of which are determined by the properties of the individual phases, following the rule of mixtures [7]. The structure of steel in the necessary place can be modified by special local treatment, where creation of strengthening or a reinforcing phase due to local phase transformation and changes of microstructure become available. The creation of structural strengthening ribs can significantly affect the overall strength and elasticity of thin-walled steel parts which have insufficient or very low bending strength and stiffness. The bending stress increases linearly away from the neutral axis until the maximum values at the top and bottom of the bent plate. Therefore, the reinforcement of metal surface layers can be an effective method of strengthening thin-walled metal structures affected by bending loads. 

Laser-assisted methods of surface treatment are one of the most popular and well-established methods of surface modification of metals. Laser treatment technologies allow local heating, high temperature gradients and cooling speeds of 105–106 °C/s due to the thermal conductivity of metal. Therefore, the hardening of low-carbon steels with less than 0.3 % carbon is also possible, whereas these steels cannot be hardened effectively by other heat treatment methods [8]. 

Transformation hardening, nitriding and carburizing by laser allows changes to the microstructure and properties such as strength, hardness, roughness, coefficient of friction, wear resistance, chemical resistance and corrosion resistance of the surface of various metals [9,10]. The typical thickness of the hardened layer after laser transformation hardening by CO_2_ laser, without melting of the surface, usually does not exceed 0.3 mm, applying a laser pulse of 0.15 mm [11]. The efficiency of laser transformation hardening and the final strength of the laser-processed structure depends on the total laser-processed area and the depth of hardening.

Therefore, application of laser transformation hardening without melting for strengthening of low-carbon steel structures with less than 0.3% carbon is limited by the low thickness of the hardened layer, because a very large surface area must be processed. In contrast, laser processing with melting gives a thicker laser-processed layer.

The main objective of this research was to test and model the bending of laser-processed steel plates, evaluating the efficiency of the application of laser treatment with surface melting to strengthening thin sheet components from unalloyed and structural carbon steels containing less than 0.2% carbon.

The results of a study of the influence of laser treatment on the microstructure and mechanical properties of bent plates of low-carbon steel are presented. The results of modelling of elastoplastic deformations of differently laser-processed samples are compared with results obtained by bending tests on real samples. The research results show that local laser treatment with surface melting can be used to increase the bending strength of thin-walled structural elements from steel (1.0402) and to decrease its deflection under identical workloads.

## 2. Object of the Study

One of the most popular grades of structural high-quality-carbon steel (1.0402) containing less than 0.3% carbon was used (Table 1 and Table 2). The microstructure and mechanical properties of this hypoeutectoid steel, according to standard EN 10250-2, can vary depending on the treatment applied [12]. Thin metal plates of size 20 mm × 150 mm × 2 mm were used for the bending test and structure analysis. 

First, before laser treatment, all the samples were subjected to tempering for internal stress relief. The microstructure of the steel (1.0402) samples after tempering demonstrated a typical hypoeutectoid ferrite-pearlite microstructure, with hardness 135 HV. The surface of the samples was subjected to additional blast cleaning to ensure uniform laser energy absorption. A “Power Plus Tools” (Shanghai, China) sandblaster and quartz sand, “Sakret” (Berlin, Germany) with a grain size of 0.1–0.5 mm were used for surface preparation until roughness Ra measured less than 5 μm. 

## 3. Research Methodology

### 3.1. Laser Surface Treatment

The Nd:YAG 4-axis laser-welding machine BMM400 (Boaolaser, Beijing, China) was used for local laser treatment. The metal samples were tightly fixed to the work table of the laser machine. A shielding gas mixture Ar-CO_2_ (20% CO_2_) with a shielding gas flow rate 20 L/min was used for Nd:YAG laser treatment. The depth of penetration should be the minimum necessary to provide sufficient hardening and avoid excessive embrittlement, cracking and deformation of the laser-processed, thin-walled steel plate [13,14]. Melting by laser or laser transformation hardening, without melting of most metal, proceeds at low energy density in the range of 10^3^–10^5^ W/cm^2^ [15]. 

One of the criteria for the selection of the optimum processing mode was the required depth of laser penetration. A depth of penetration of about 0.35 mm, amounting to 20% of the total thickness of the metal plate, was used in the present experiments. The width of the track of the laser-processed metal was approximately 0.7 mm. The most appropriate laser processing parameters were calculated, using the methodology presented in reference literature [15], considering the physical properties of the steel (Table 3) and the technical characteristics of the 400 W laser equipment. The laser processing parameters, other than the number of processed sides, number of laser tracks and distance between them, remained constant throughout all experiments (Table 4).

The dimension of the laser-processed surface area in all cases was 40 mm × 20 mm. Four different laser-processing cases were adopted that differed in the number of laser tracks. In case I, no laser tracks were made at all, i.e., the metal plate was left unprocessed. In case II, 38 laser tracks were made, with the tracks overlapping in approximately 30% of their width. In case III, 17 laser tracks were made, with a distance between the tracks approximately equal to 50% of their widths. In case IV, 14 laser tracks were made, with a distance between the tracks equal to the track width. 

**Figure 1:**
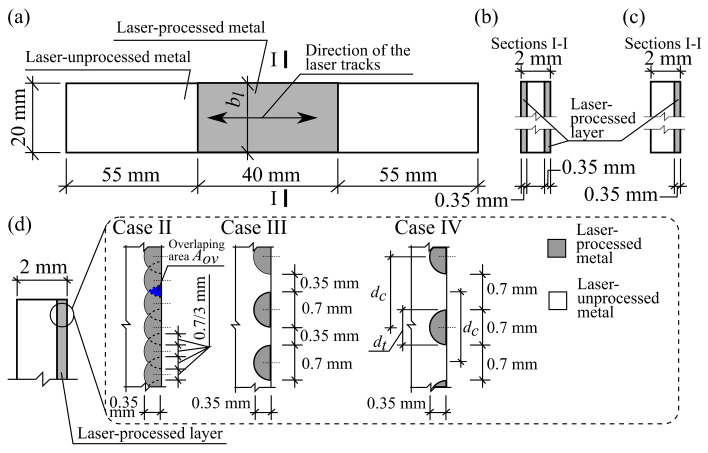
The general view of the plate specimen (**a**); sketches of their cross-sections: of the one-side laser-processed plate (**b**), and of the two-side laser-processed plate (**c**); and cases of the laser tracks (**d**).

All four cases were applied to both one-side-processed and two-side-processed plates. In case II, case III and case IV, the laser-processed tracks were oriented along the plate, in the direction in which the greatest stresses appear, by analogy with the principle of the strengthening of composite materials. A general view of the plate specimen and the laser-treated area is shown in Figure 1a. The plate cross-sections and the laser-processed layers are shown in Figure 1b,c. The laser processing cases, track width, depth and positions are depicted in Figure 1d.

### 3.2. Experimental Methods of Investigation of the Materials Properties and the Bending Stiffness

The mechanical properties of the base, i.e., laser-unprocessed metal, i.e., stress-strain diagram, modulus of elasticity $E_{b}$, offset yield stress (proof stress) $\sigma_{0.2,B}$, ultimate and fracture stresses as well as the fracture strains $\epsilon_{ul,b}$, were determined by the standard tensile test according to LST EN ISO 6892-1 [17]. The corresponding mechanical properties of the laser-processed metal, i.e., $E_{l}$, $\sigma_{0.2,l}$ and $\sigma_{0.2,B}$ were determined indirectly by using the empirical relationships between the corresponding mechanical properties and hardness which was determined according to EN ISO 6507-1 [18]. The series of three samples were used for the mechanical tests—the estimated means $m_{x}=\sum_{i=1}^{N}x_{i}$ are presented hereafter and were used for the calculations presented below. Unmachined samples of type B, with initial gauge length $L_{0}=80$ mm, length $L_{c}=120$ mm and initial cross-section $S_{0}=40$ mm^2^ were used for the tensile test. 

A universal tensile-testing machine TIRAtest 2300 (TIRA, Schalkau, Germany) with a bending test tool and Catman-Express software (version 5.1, HBM, Germany) was used for the tensile and bend tests. A tension dynamometer up to 50 kN was used for the tensile test, while a compression dynamometer up to 1 kN was used for the bending test, described in the last paragraph of this subchapter. Steel hardness was determined using a Zwick/Roell ZHU (Ulm, Germany) universal hardness tester using the Vickers method. Steel hardness was determined on the surface with a load of 10 N. The hardness of the laser-processed layer was determined using a Zwick/Roell ZHμ (Ulm, Germany) hardness tester with a diamond, square-based tetrahedral pyramid tip with a load of 2.942 N. Hardness was measured on the surface of the laser-processed layers and on its cross-section. 

The chemical composition of the steel was determined using the PMI Master PRO Oxford Instruments (High Wycombe, UK) optical emission spectrometer. 

The metallographic examination of the steel and the laser-processed layer and analysis of the geometry and dimensions of the laser-processed layer was carried out using a Nikon Eclipse MA200 optical microscope (Tokyo, Japan) with a Lumenera Infinity 2-2 video camera and a JEOL JSM-7600 (Tokyo, Japan) scanning microscope with an energy dispersive spectrometer (EDS) Oxford INCA Energy X-Max20 (Oxford, UK) at different magnifications (up to ×1500). 

The qualitative X-ray diffraction (XRD) analysis of the phase composition of materials was carried out by applying diffractometer DRON-7 (Burevestnik, Saint-Petersburg, Russia). Graphite-monochromated Cu Kα radiation (λ  =  0.154178 nm) was used. The parameters of the tests were as follows: voltage—30 kV; current—12 mA; the range of the diffraction angle—from 4° to 120°, with detector movement steps of 0.04°; the duration of the intensity measuring in each step—2.0 s.

**Figure 2:**
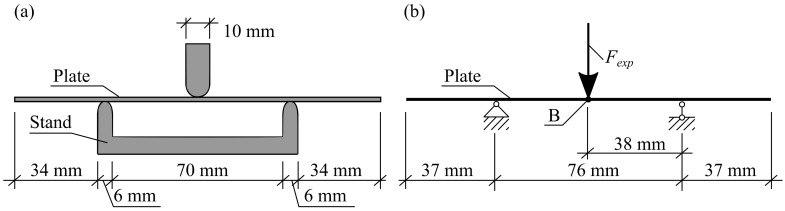
The principal view of the bending test device (**a**) and corresponding calculation scheme (**b**).

The elastoplastic bending of the thin plate was conducted by using a 3-point bending device (see Figure 2), analogous to the device used in the metal bending tests, according to LST EN ISO 7438 [19]. The calculation scheme of the bending test is shown in Figure 2b. The distance between supports was 76 mm, the rounding radius and the Poisson’s width were 10 mm. The test was conducted by using the strain-control mode with a velocity of displacement of 1.5 mm/s. The load $F_{exp}$ was imposed at the middle of the span, at point B; see Figure 2b. The load $F_{exp}$ ranged from 0 N up to the maximum load, corresponding to the greatest deflections of the experiment, which equal 2 mm. In general, $F_{exp}<500$ N. The deflections were measured at the middle of the span of the plate, i.e., at point B; see Figure 2b. It should be noted that the distance between the supports, 76 mm, was greater than required according to LST EN ISO 7438 [19] to decrease the influence of shear strains on the deflections.

In the case of bending the one-side laser-processed plate, the laser-processed layer may be under tension or under compression. To evaluate the influence of the stress-strain state of the laser-processed layer on the deflections or the bending stiffness of the plate, 3 different loading variants were considered. In Variant A, the bending load $F_{exp}$ was imposed on the laser-processed side of the one-side laser-processed plate, so that the laser-processed layer was under compression. In Variant B, $F_{exp}$ was imposed on the laser-unprocessed side of the one-side laser-processed plate, so that the laser-processed layer was under tension. In Variant C, $F_{exp}$ was imposed on the two-side laser-processed plate. In this case, the top laser-processed layer was under compression, while the bottom laser-processed layer was under tension. Also, 4 already mentioned laser-processing cases were considered with each loading variant: case I, laser-unprocessed plate; case II, 38 laser tracks; case III, 17 laser tracks; and finally, case IV, 14 laser tracks. In total, 10 different force–deflection experiment cases were conducted. These cases are summarized in Table 5. In general, three measurements were performed to determine each quantity. Three plates were tested for case I, and 9 plates for the rest of the laser-processed plate cases: case II–case IV. Thus, in general, 39 plates were tested for the bending experiment. The estimated mean values $m_{x}=\sum_{i=1}^{N}x_{i}$ are presented and analyses hereafter are in the present article.

### 3.3. Finite Element Analysis (FEA) Simulation of Bending of Thin Metal Plate

**Figure 3:**
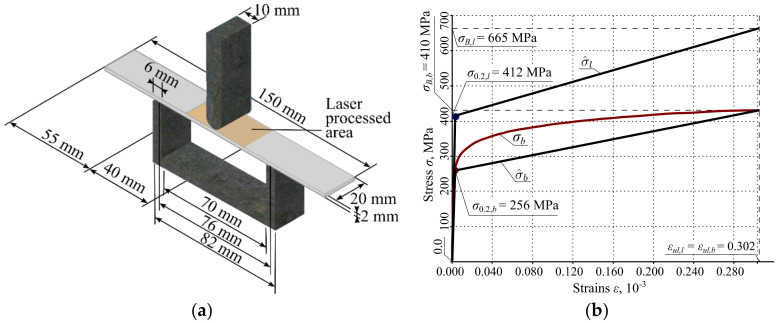
Part (**a**) shows: geometry, dimensions, stand and the loading scheme of the laser-processed plate of the numerical model of the bending simulation; and in (**b**): $\sigma_{b}$ denotes the base metal σ−ϵ curve; ${\hat{\sigma }}_{b}$ denotes the bilinear approximations of $\sigma_{b}$; ${\hat{\sigma }}_{l}$ denotes the bilinear approximation of the σ−ϵ curve of unknown laser-processed metal; yield strengths, ultimate strength, and fracture strains of the base metal and the laser-processed metal are denoted by $\sigma_{0.2,b}$, $\sigma_{0.2,l}$, $\sigma_{B,b}$, $\sigma_{B,l},$ $\epsilon_{ul,b}$ and $\epsilon_{ul,l}$ respectively.

The bending of the thin metal plate was modeled by finite element analysis (FEA), using the Ansys workbench software package, version 16.0. A numerical model of the bending test was created identical to the experimental bend test device; see sub-chapter 3.2. The general view of the numerical model is shown in Figure 3a. The geometry and dimensions of the FEA models of the plate were identical to the plates used in the experimental investigation; see Figure 1. The shape and depth of the laser-processed metal (0.35 mm) were identical for all FEA models of the plates (see Figure 3 and Table 6). The distance between supports (76 mm), punch width (10 mm) and fixation conditions in the FEA simulation were the same as those used during the real experiments. The plates in FEA modeling were supported simply, as it is shown in Figure 2b. For all three variants, the load was imposed in the middle of the span of the stand; see Figure 3a. The laser processing cases and loading variants used in the present FEA are summarized in Table 6. As in the case of experimental investigation in the numerical modeling in general, 10 different simulations were conducted; see Table 6.

The three-dimensional solid brick and tetrahedral elements were used for the discretization of the complex geometry of the modelled plate with laser-processed layers [20]. Large-scale finite elements with a maximum size of 0.7 mm were used to mesh the laser-unprocessed parts of the plate, while a finer mesh, with finite element sizes up to 0.12 mm, was adopted for the discretization of the laser-processed layer. 

The different mechanical properties of the base metal and laser-processed layer were used in the FEA models (treatment cases IIA—IIC, IIIA—IIIC, IVA—IVC). The yield strength, ultimate strength and modulus of elasticity of the steel were determined by mechanical tensile and bending testing of the specimens. The approximate yield strength and ultimate strength of the metal of the laser-processed layers were obtained by applying the relations between hardness and strength that are explained in [21,22,23]. The modulus of elasticity, Poisson’s ratio and other required properties of materials are taken from reference literature [5]. The bilinear isotropic hardening plasticity model was used for the bending case numerical investigation [8].

The so-called bilinear constitutive laws of the laser-unprocessed and laser-processed metals of the plates were adopted for the finite element analysis of the bending of the plates; see ${\hat{\sigma }}_{b}$ and ${\hat{\sigma }}_{l}$ curves depicted in Figure 3b. These constitutive laws are approximations of the real physical stress-strain (σ−ϵ) curves of the laser-processed and unprocessed metals. The direct investigation of the σ−ϵ curve of the laser-processed metal is very complicated; therefore, the simplified bilinear σ−ϵ diagrams already mentioned were adopted for the finite element analysis. Below, explanations are given of the obtained and adopted mechanical parameters of the laser-unprocessed and processed metals of the plates.

The σ−ϵ curve of the base, i.e., laser-unprocessed, metal was obtained experimentally, as described above in Sub-Chapter 3.2. From this σ−ϵ curve, the yield strength $\sigma_{0.2,b}=256$ MPa, the ultimate strength $\sigma_{B,b}=410$ MPa and the ultimate strains $\epsilon_{ul,b}=0.302$ of the base metal were obtained directly. The σ−ϵ curve, denoted as $\sigma_{b}$, is depicted as a smooth curve in Figure 3b. 

As can be seen from Figure 3b, the σ−ϵ curve does not exhibit a clear yield plateau. After reaching the yield strength, so-called work hardening is specific to the base metal. Due to the difficulty of experimental investigation of the mechanical properties of the laser-processed metal, the σ−ϵ curve of this metal is unknown. Only indirect estimations of the yield strength, $\sigma_{0.2,l}=412$ MPa, and the ultimate strength, $\sigma_{B,l}=665$ MPa, were obtained, using the J. R. Cahoon equations, [21,22], which establish the relations between the hardness and strength of metals. However, the ultimate strains, $\epsilon_{ul,l}$ of the laser-processed metal are still unknown. Therefore, it is assumed that the ultimate strains of the laser-processed metal are the same as for the laser-unprocessed metal, i.e., it is assumed that $\epsilon_{ul,l}=\epsilon_{ul,b}=0.302$. 

The moduli of elasticity, $E_{b}=200$ GPa and $E_{l}=210$ GPa, Poisson’s ratios $v_{b}=v_{l}=0.28$ and the shear moduli, $G_{b}=78.1$ GPa and $G_{l}=82$ GPa of the base metal and the laser-processed metal respectively, were taken from the reference literature [23]. Having the yield strength, $\sigma_{0.2,i}$, and the moduli of elasticity, $E_{i}$, $i\in \left\{b,l\right\}$, the strains $\epsilon_{1,b}=1.28\cdot 10^{-3}$ and $\epsilon_{1,l}=1.962\cdot 10^{-3}$ that correspond to the yield strength $\sigma_{0.2,i}$ were calculated by the formula $\epsilon_{1,i}=\sigma_{0.2,i}/E_{i}$. 

The strength coefficients of the laser-processed and unprocessed metals, $E_{1,b}=512$ MPa and $E_{1,l}=843$ MPa, respectively, were calculated by the formula $E_{1,i}=\left(\sigma_{B,i}-\sigma_{0.2,i}\right)/\left(\epsilon_{ul,i}-\epsilon_{1,i}\right)$, $i\in \left\{b,l\right\}$, where $\sigma_{0.2,b}=256$ MPa and $\sigma_{0.2,l}=412$ MPa are the yield strengths of the base metal and the laser-processed metal, respectively, while $\sigma_{B,b}=410$ MPa and $\sigma_{B,l}=665$ MPa are the ultimate strengths of the same metals. 

On the basis of the obtained values of the mechanical properties of the metals, the bilinear approximations of σ−ϵ curves of the laser-unprocessed and processed metals ${\hat{\sigma }}_{b}$. and ${\hat{\sigma }}_{l}$, respectively, are depicted in Figure 3b. All the values of the mechanical properties of the two metals discussed above are summarized in Table 7.

### 3.4. Analytical Analysis of the Stiffness Properties of the Laser-Unprocessed and Processed Plates

The geometry and dimensions of the modeled plate are given in Figure 1. The material properties are given in Table 7. The main assumptions are as follows: (1) the laser-processed metal has a perfect bond with the base metal; that is, no slip occurs between the laser-processed layer and the layer of the base metal; (2) the hypotheses of the plane sections are valid; (3) the influence of Poisson’s ratio is ignored; (4) only the normal stresses σ acting along the laser tracks (see Figure 1a) are taken into account; (5) the stress-strain diagram for the laser-processed layer and the base metal is bilinear, as shown in Figure 3b.

Then, under the accepted assumptions, we can write that the total axial force acting on the cross-section of the plate, $N\left(\epsilon \right)={Ν}_{b}\left(\epsilon \right)+{Ν}_{l}\left(\epsilon \right)$; where ${Ν}_{b}\left(\epsilon \right)=A_{b}{\hat{\sigma }}_{b}\left(\epsilon \right)$ and ${Ν}_{l}\left(\epsilon \right)=A_{l}{\hat{\sigma }}_{l}\left(\epsilon \right)$ are the axial forces acting in the base metal and laser-processed metal of the plate, respectively; $A_{b}$ and $A_{l}$ are the cross-sectional areas of the base metal and the laser-processed metal, respectively; and ${\hat{\sigma }}_{l}$ are the stress functions of the base and the laser-processed metals, respectively, depending on the strains, ϵ. 

When the bilinear stress–strain diagrams are applied (see Figure 3b), then these functions ${\hat{\sigma }}_{b}$ and ${\hat{\sigma }}_{l}$ can be expressed as follows:

(1) $${\hat{\sigma }}_{i}\left(\epsilon \right)=\left\{\begin{array}{c} E_{i}\epsilon ,\;\mathrm{as}\;\epsilon \leq \epsilon_{1,i},\\ E_{i}\epsilon_{1,i}+E_{1,i}\left(\epsilon -\epsilon_{1,i}\right),\;\mathrm{as}\;\epsilon_{1,i}<\epsilon \leq \epsilon_{ul,i},\;i\in \left\{b,l\right\},\\ 0,\;\mathrm{as}\;\epsilon >\epsilon_{ul,i}. \end{array}\right.$$

where $E_{b}$, $E_{1,b}$, $E_{l}$, $E_{1,l}$, $\epsilon_{ul,b}$ and $\epsilon_{ul,l}$ are the moduli of elasticity, the stiffness moduli and the ultimate strains of the base and the laser-processed metals, respectively; see Table 7 and Figure 3b. 

Since $\epsilon_{1,b}\leq \epsilon_{1,l}$ and $\epsilon_{ul,b}\leq \epsilon_{ul,l}$, then by putting functions ${\hat{\sigma }}_{b}$ and ${\hat{\sigma }}_{l}$ (see Equation (1)), in $N\left(\epsilon \right)=\sum_{i\in \left\{b,l\right\}}A_{i}{\hat{\sigma }}_{i}\left(\epsilon \right)$, we obtain an explicit function for the axial force, N:

(2) $$N\left(\epsilon \right)=\left\{\begin{array}{c} \epsilon \sum_{i\in \left\{l,b\right\}}A_{i}E_{i},\;\mathrm{as}\;\epsilon \leq \epsilon_{1,b},\\ A_{b}\left(E_{b}\epsilon_{1,b}+E_{1,b}\left(\epsilon -\epsilon_{1,b}\right)\right)+A_{l}E_{l}\epsilon ,\;\mathrm{as}\;\epsilon_{1,b}<\epsilon \leq \epsilon_{1,l},\\ \sum_{i\in \left\{l,b\right\}}A_{i}\left(E_{i}\epsilon_{1,i}+E_{1,i}\left(\epsilon -\epsilon_{1,i}\right)\right),\;\mathrm{as}\;\epsilon_{1,l}<\epsilon \leq \epsilon_{ul,b},\\ A_{l}\left(E_{l}\epsilon_{1,l}+E_{1,l}\left(\epsilon -\epsilon_{1,l}\right)\right),\;\mathrm{as}\;\epsilon_{ul,b}<\epsilon \leq \epsilon_{ul,l},\\ 0,\;\mathrm{as}\;\epsilon >\epsilon_{ul,l}. \end{array}\right.$$

### 3.5. Evaluation of the Cross-Sectional Area of Laser-Processed Metal Track

**Figure 4:**
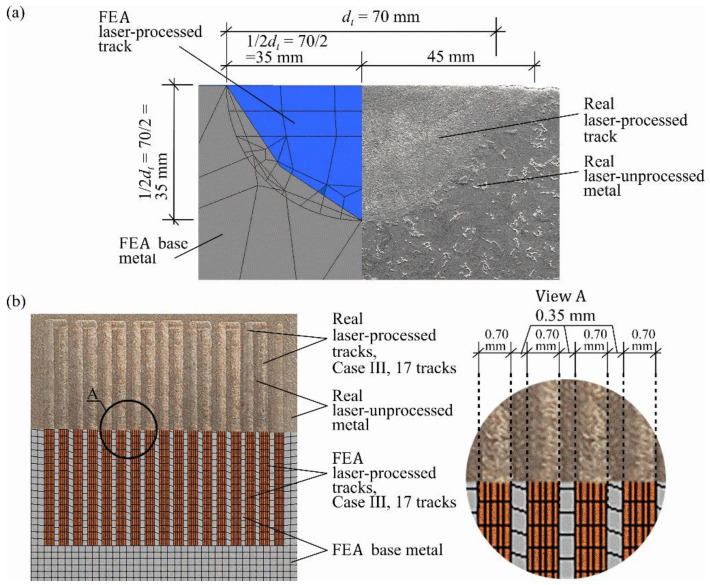
Cross-sections of a laser-processed track and the base metal of a FEA and a real specimen respectively (**a**) and the general view of the laser-processed tracks of case III of the real specimen and its FEA model respectively (**b**).

The geometry of the laser-processed layer is not rectangular, but of more difficult geometry, as shown in Figure 1. Therefore, it is worth discussing further the evaluation of the area of the cross-section of the laser-processed metal, $A_{l}$. As can be seen from Figure 1d and Figure 4, the cross-sections of the laser-processed layer and a single laser-processed track are not rectangular. If the laser tracks do not overlap, i.e., $d_{c}\geq d_{t}$, where $d_{t}$ is the laser-processed track width and $d_{c}$ is the distance between the tracks of the laser-processed metal (see Figure 1d, case IV), and the number of laser tracks, $n_{tr}$, is known, then the cross-section of the laser-processed metal can be calculated easily.

(3) $$A_{l}=n_{s}n_{tr}A_{tr,1},\;\mathrm{if}\;d_{c}-d_{t}\geq 0,\;\mathrm{and}\;n_{tr}\;\mathrm{is}\;\mathrm{known}$$

where $A_{tr,1}=\pi d_{t}^{2}/8$ is the cross-sectional area of one laser-processed track, assumed to be a semicircle; $n_{s}\in \left\{1,2\right\}$ is the number of laser-processed sides of the plate. It should be noted that, in Equation (3) and hereafter in the article, the number of laser tracks, $n_{tr}$, may not be an integer; i.e., in general, $n_{tr}\in \mathbb{R}$.

However, if $n_{tr}$ is not known or the laser-processed tracks overlap, i.e., $d_{c}<d_{t}$, then the exact evaluation of $A_{l}$ can be complicated. Some suggestions are made below on the following assumptions: the cross-section of a laser track is a semicircle whose area $A_{tr,1}=\pi d_{t}^{2}/8$, where $d_{t}$ is the width of the laser track (see Figure 1d); the width of the tracks, $d_{t}$, is constant for all laser tracks and for the entire length of all laser tracks; the distance between centers of the laser tracks, $d_{c}$ (see Figure 1d) is also constant for all pairs of the vicinal laser tracks.

The calculation of $A_{l}$ depends on the distances between the tracks, $d_{c}$. If the difference $d_{c}-d_{t}>0$ then the laser tracks do not overlap each other and, in general, the exact evaluation of $A_{l}$ is impossible without knowledge of the exact position of the laser tracks. However, the lower and upper bounds $A_{l,inf}$ and $A_{l,sup}$ can be suggested to estimate $A_{l}$, $A_{l,inf}\leq A_{l}\leq A_{l,sup}$:

(4) $$A_{l,inf}=n_{s}A_{tr,1}floor\left(b_{l}/d_{c}\right),\;\mathrm{if}\;d_{c}-d_{t}>0,$$

(5) $$A_{l,sup}=n_{s}A_{tr,1}ceil\left(b_{l}/d_{c}\right),\;\mathrm{if}\;d_{c}-d_{t}>0.$$

where $floor\left(x\right)=max\left\{y\in \mathbb{Z},\;y\leq x\right\}$ and $ceil\left(x\right)=min\left\{y\in \mathbb{Z},\;y\geq x\right\}$ are ceiling and floor functions; ℤ is the set of the integer numbers; $b_{l}$ is the width of the laser-processed area. When $b_{l}/d_{c}$ increases, then the relative differences of the estimations $\left(A_{l,sup}-A_{l,inf}\right)/A_{l,sup}$ and $\left(A_{l,inf}-A_{l,sup}\right)/A_{l,sup}$ decreases. For the present case, when $d_{t}=0.7$ mm, and $d_{c}\in \left\{1.05,\;1.4\right\}$ mm (see Figure 1d), Equations (3) and (4) give: $A_{l,inf}=14A_{tr,1}$ and $A_{l,sup}=15A_{tr,1}$ when $d_{c}=1.4$ mm, and $A_{l,inf}=19A_{tr,1}$ and $A_{l,sup}=20A_{tr,1}$ when $d_{c}=1.05$ mm. 

If $n_{tr}$ is unknown, then it can be estimated as follows:

(6) $${\hat{n}}_{tr}=b_{l}/d_{c}$$

It is clear that Equation (6) can be used to evaluate the distance between the laser-processed tracks $d_{c}=b_{l}/{\hat{n}}_{tr}$. 

When $d_{c}-d_{t}=0$ and $n_{tr}$ is known, then $A_{l}$ can be calculated by Equation (3). Otherwise, when the number of laser-processed tracks, $n_{tr}$ is not known, then $A_{l}$ can be calculated by assuming that there are no laser-unprocessed bands between the laser-processed tracks by the following formulae:

(7) $$A_{l}=n_{s}A_{tr,1}b_{l}/d_{c},\;\mathrm{if}\;d_{c}-d_{t}=0.$$

When $d_{c}-d_{t}<0$, then the laser tracks overlap each other and we have to take into account the overlapped areas of the tracks, see Figure 1d, case II. Therefore, Equations (3)–(5) and (7) are not valid. On the basis of the above assumptions, the following bounds infimum $A_{l,inf}$ and supremum $A_{l,sup}$, $A_{l,inf}\leq A_{l}\leq A_{l,sup}$, of the cross-section area $A_{l}$ of the laser-processed metal are derived when $n_{tr}$ is not known

(8) $$A_{l,inf}=n_{s}{\hat{n}}_{tr}(A_{tr,1}-A_{ov}),\;\mathrm{if}\;d_{c}-d_{t}<0,$$

(9) $$A_{l,sup}=\min\{n_{s}({\hat{n}}_{tr}A_{tr,1}-\left({\hat{n}}_{tr}-1\right)A_{ov}),b_{l}1/2d_{t}\},\;\mathrm{if}\;d_{c}-d_{t}<0,$$

where ${\hat{n}}_{tr}$ is an estimation of the number of laser-processed tracks:

(10) $${\hat{n}}_{tr}=floor\left(\frac{b_{l}}{d_{c}}\right)-1+\left(1+frac\left(\frac{b_{l}}{d_{c}}\right)\right)\frac{d_{c}}{d_{t}}$$

where $frac\left(x\right)=x-floor\left(x\right)$ is the fractional part of a number x.

When the number of the tracks $n_{tr}$ is known:

(11) $$A_{l}=n_{s}(n_{tr}A_{tr,1}-\left(n_{tr}-1\right)A_{ov}),\;\mathrm{if}\;d_{c}-d_{t}<0\;\mathrm{and}\;n_{tr}\;\mathrm{is}\;\mathrm{known}$$

In Equations (8), (9) and (11), $A_{ov}$ is the overlapping area of two adjacent laser tracks; see Figure 1d, case II. Under the accepted assumptions, $A_{ov}$ can be calculated as the area of the circle segment $A_{ov}=d_{t}^{2}\left(\alpha -\sin\left(\alpha \right)\right)/8$, where $\alpha =2\tan^{-1}\left(\sqrt{d_{t}^{2}-d_{c}^{2}}/d_{c}\right)$ is the sector angle, $\tan^{-1}$ is the inverse tangent function. It should be noted that $A_{l,inf}\leq A_{l,sup}\leq A_{l,upp}=1/2\;b_{l}d_{t}$, where $A_{l,upp}$ is the upper bound of the cross-section of the laser-processed layer.

## 4. Results

The results of the structural analysis and hardness of the laser-processed surface layer are given in Section 4.1. The comparative analyses of the experimental and numerical FEA results of the bending of the laser-unprocessed (case I) and laser-processed plates are given in the Section 4.2 and Section 4.3. In Section 4.2 the numerically obtained von Mises stresses and forces $F_{exp}$ at the different vertical displacements $w\in \left\{0.5,\;1.0,\;1.5,\;2.0\right\}\;\mathrm{mm}$ imposed at midpoint B are presented, see Figure 2b. The relation between the experimentally determined vertical forces $F_{exp}$, imposed at the midpoint B, and the deflection w is analyzed in Section 4.3. Furthermore, a comparison of the experimental and calculated-by-FEA vertical forces $F_{exp}$ and $F_{calc}$ is given in Section 4.3. In Section 4.4.1, the results of the analytical analysis of the cross-section areas $A_{l}$ of the laser-processed metal depending on the track width of the laser-processed metal $d_{t}$ and the distances between these tracks centers $d_{c}$ are given. In Section 4.4.2, the results of the analytical analysis of the influence of the laser processing of the plate metal on the axial stiffness of the cross-section and the force-strain behavior of the plates under investigation are given. The obtained results definitely showed that the axial and flexural stiffnesses of the laser-processed cross-sections are bigger than the stiffnesses of the laser-unprocessed cross-sections.

### 4.1. Results of the Structural Analysis of the Laser-Processed Surface Layer 

**Figure 5:**
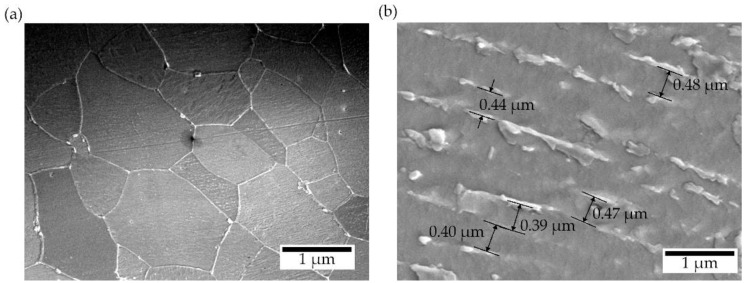
SEM images of microstructure: (**a**) ferrite-pearlite structure of base metal (magnification ×2000); (**b**) distance between lamella in the sorbite structure of laser-processed layer (magnification ×10000).

**Figure 6:**
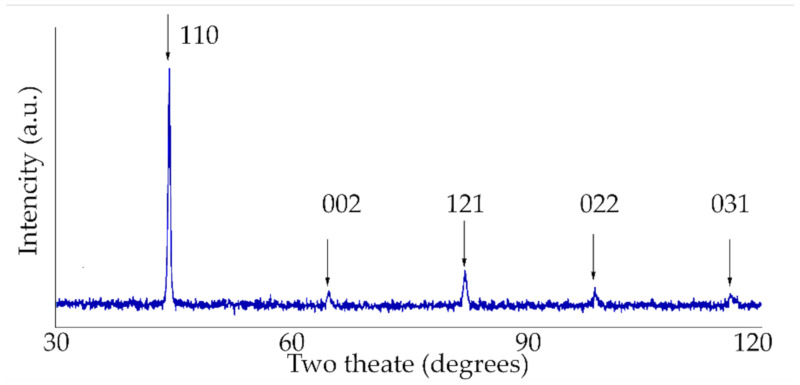
XRD pattern of the laser-processed layer.

**Figure 7:**
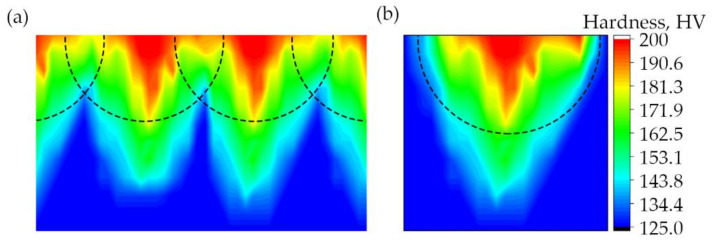
Distribution of hardness through the cross-section of the laser-processed surface of sample IIA: (**a**) and (**b**) show the distributions of three- and one-laser-processed tracks respectively.

The thickness of the laser-processed layer, which is established by metallographic examination of cross-sections of samples of metal processed by laser, was about 0.35 mm (see Figure 4). There are no unacceptable inclusions, porosity or internal defects in the remelted area and transition area. The microstructure of the base metal consists of 70% ferrite and 30% pearlite (see Figure 5a), according to GOST 8233 [26]. Granularity in the processed zone decreases from G8 (average grain diameter about 18 µm) to G10 (average grain diameter about 10 µm), according to ISO 643 [27]. According to the XRD data analysis (see Figure 6), the X-ray diffraction pattern of the laser-processed layer is typical for the ferrite microstructure family (including sorbite and troostite) with BCC crystal structure). The same crystal structure has martensite and bainite. The Fe_3_C peak and other carbide peaks were not observed. This is typical, so carbon content in the steel is low or peak sensitivity is below the limit of detection sensitivity. Therefore, XRD analysis confirms only that there is no unstable, retained austenite in the laser-processed area, because the austenite has the FCC crystal structure and other XRD diffraction peaks. In low-carbon steel, high-retained austenite contents are usually found together with the martensite or bainite phase in the quenched steel after cooling. The measurement of microhardness shows that the hardness of the laser-processed layer increases up to 200 HV (by 60%) compared to the laser-unprocessed base metal hardness (see Figure 7). According to the hardness measurement results, there are no hard and brittle bainite and martensite microstructures in the laser-processed areas or their proportion is very low, because such quenching microstructures have the highest hardness: bainite—about 400 HV and martensite—above 450 HV [28,29]. Consequently, to achieve a hardness of about 200 HV, it is necessary to have a minimum of about 25% of bainite or martensite structure in the present low-carbon steel after rapid cooling of the microstructure of the melted area, or the steel’s microstructure must be strengthened by refining the grain size and creating a finely dispersed perlite (sorbite, troostite) structure. 

The microstructure located at the laser-processed area of the low-carbon steel sample demonstrated a typical sorbite structure (Figure 5b). The distance between lamella, which is measured by SEM, is less than 0.3–0.4 µm. This distance is typical for sorbite, because perlite has a distance between lamella of about 0.6–0.7 µm, troostite—about 0.1 µm, martensite and bainite—about 0.2 µm and thickness of retained austenite lamellae—0.05–0.2 µm [30]. The hardness of the laser-processed layer also corresponds to the typical hardness of sorbite, which must be in the range 200–300 HV [31].

The sorbite structure of the laser-processed layer was formed due to the applied high overlap coefficient of laser spots, with inevitable additional heating and partial remelting of the crystallized pool during the next laser pulse. This effect allows the cooling rate of the melted pool to be reduced and prevents the formation of more brittle, quenched structures in the laser-processed layer.

**Figure 8:**
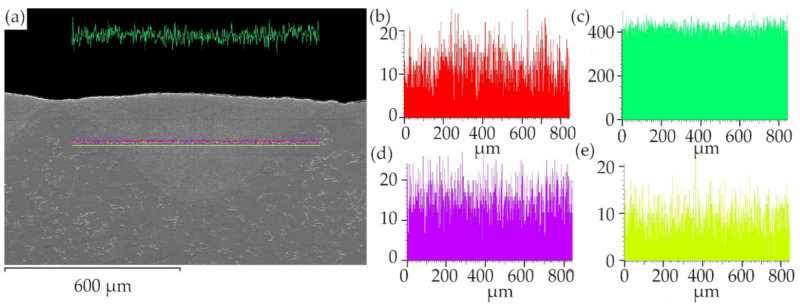
Line scan element analysis along the surface of laser-processed layer: (**a**) place of line scan in cross-section of layer; (**b**) C distribution; (**c**) Fe distribution; (**d**) Si distribution; (**e**) Mn distribution.

**Figure 9:**
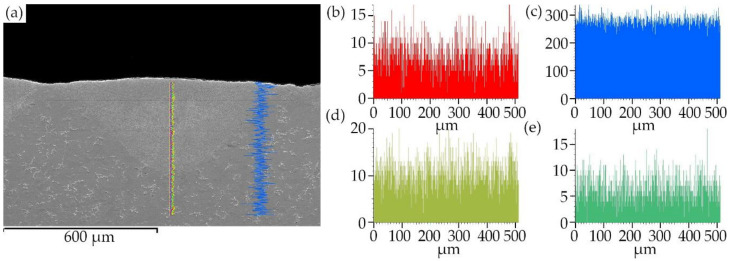
Line scan element analysis normal to the laser-processed surface: (**a**) place of line scan in cross-section of layer; (**b**) C distribution; (**c**) Fe distribution; (**d**) Si distribution; (**e**) Mn distribution.

The sorbite structure has many advantages in this case, because sorbite has a finer texture, with higher dispersity and stiffness than pearlite, which increases the strength and wear resistance of the laser-processed metal parts, without loss in plasticity, that is typical for hard and brittle quenching (martensite or bainite) microstructures [32]. SEM-EDS element mapping, line scan and point analysis of the distribution and concentration of chemical elements (C, Mn, Si) in the remelted area show that there is no significant chemical heterogeneity in the laser-processed layer (see Figure 8 and Figure 9, Table 8). This uniform distribution of the chemical elements and the homogeneous structure of the remelted layer can positively influence the mechanical properties of the laser-processed layers and the entire plate.

### 4.2. Results of the FEA Simulations of the Bending of the Laser-processed Plates

The increase in the bending force varied, depending on the plate treatment case and deflection level: for 0.5 mm deflection, the force was 11–27%; for 1.0 mm deflection about 43–49%; for 1.5 mm deflection—32–55%; for 2.0 mm deflection—30–52%. The increase in the maximum equivalent stresses varied according to the plate treatment case and deflection level: for 0.5 mm deflection—13–30%; for 1.0 mm deflection—13–32%; for 1.5 mm deflection—8–26%; for 2.0 mm deflection—8–25%. 

The maximum equivalent stress at the bent plates depends on the volume and position of the laser-processed layer. The greatest increase in the required bending load and available stresses, according to the modeling results, was in the double-sided laser-processed samples, while the volume of treatment was greatest from 15.4% to 19.8% of the volume of the bent plate part. The increased bending load required for similar deflection depends also on the position of the laser-processed layer. Better results were obtained in the bent samples where the zone of the laser-processed layer was subjected to tension, rather than compression. 

**Figure 10:**
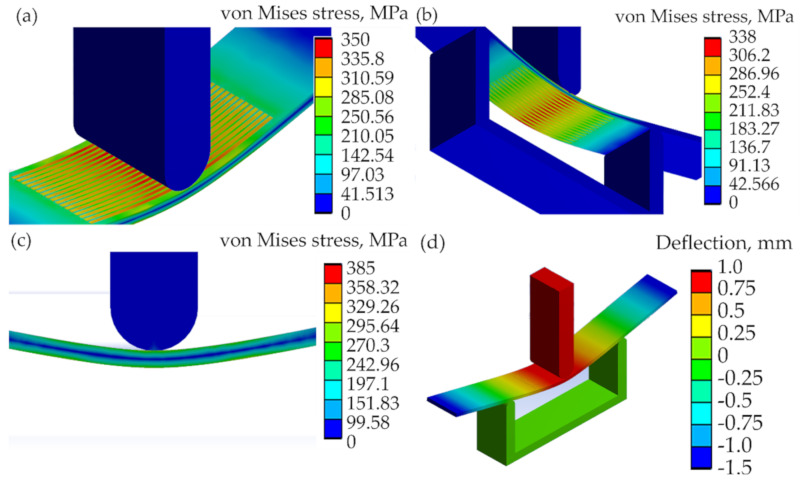
FEA results of the bending modeling of the laser-processed plates when the imposed vertical displacement of the plate middle point B, see Figure 2b, equals 1 mm; von Mises stresses are shown in (**a**–**c**): (**a**) for case IIIA, (**b**) for case IVB and (**c**) for case IIC; while (**d**) shows the deflections or vertical displacements of the plate of case IIIA.

It was determined that double-side laser-processed metal plates with 30% overlay of laser tracks had the highest resistance to bending, compared to the other treatment modes; see Table 9. However, modeling results (see Table 9 and Figure 10) show that it is possible to use a non-continuous laser with either one-side or double-side processing with a certain distance between tracks, because the difference in the efficiency of such surface treatments is small. The difference of the required bending loads for one deflection level of the plate, applied to different positions of the laser-processed layer (cases A, B, C) did not reach 20%.

### 4.3. Commparison of the Experimental and Modeling Results of the Bending of the Laser-Processed and Unprocessed Plates

**Figure 11:**
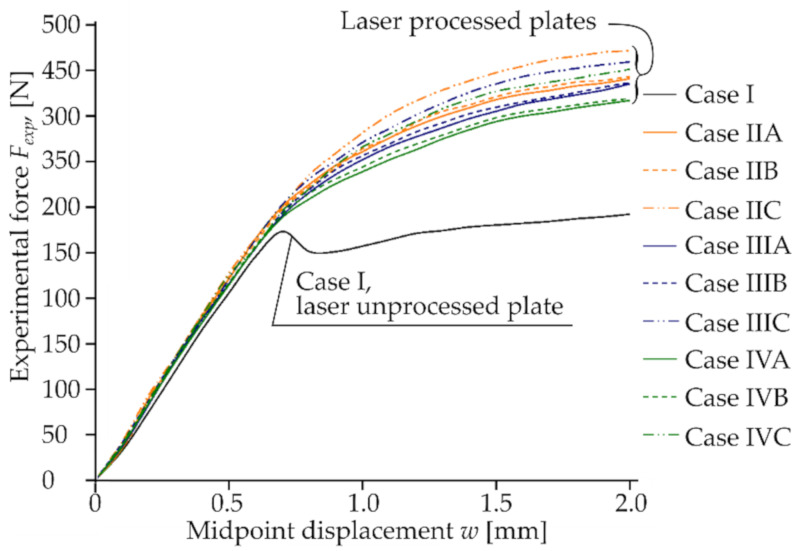
Experimental bending force $F_{exp}$ vs. deflections w of the middle point of the differently laser-processed plates.

The mechanical bend tests showed that local laser processing increases the load required to reach the same level of deflection of laser-processed samples compared to unprocessed ones; see Figure 11 and Table 10. The experimental bending load, $F_{exp}$, increases depending on the laser-processed case: for 0.5 mm deflection, the $F_{exp}$ increases from 3% up to 27%; for 1.0 mm deflection, $F_{exp}$ increases from 29% up to 48%; for 1.5 mm deflection, $F_{exp}$ increases from 32% up to 55%; for 2.0 mm deflection, $F_{exp}$ increases from 30% up to 52%. 

Experimental data confirmed that the effect of strengthening and total resistance of samples to bending was influenced by the position of the laser-processed area, the distance between laser tracks, the volume of the hardened phase and its ratio to the volume of laser-untreated material in the area of maximum stress from the applied load. The difference between modeling and bending test results was less than 15% (Table 10).

### 4.4. Results of the Analysis of the Axial Stiffness of the Laser-Processed Plate

The analysis of the results is presented mainly in terms of the relative quantities to make the analysis, discussion and results valid, not only for the particular cases considered in the present article, but also to extend the results and conclusions to other cases.

#### 4.4.1. Area $A_{l}$ of the Laser-Processed Metal

**Figure 12:**
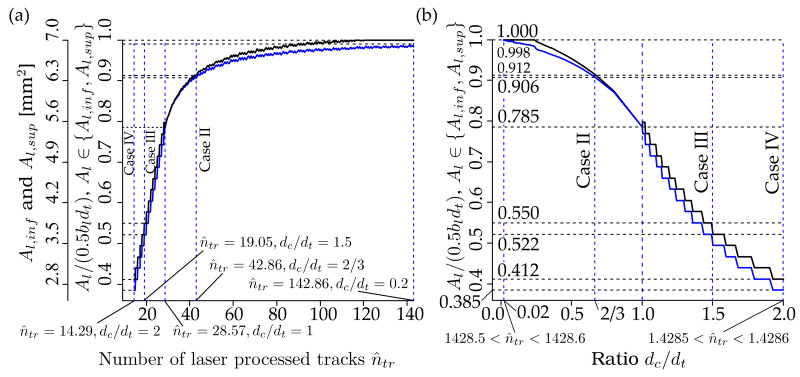
The dependencies of the bounds of the cross-section of the laser-processed metal $A_{l,inf}$ and $A_{l,sup}$ and the ratios $A_{l,inf}/\left(1/2b_{l}d_{t}\right)$ and $A_{l,sup}/\left(0.5b_{l}d_{t}\right)$ on the number of tracks ${\hat{n}}_{tr}$ and ratio $d_{c}/d_{t}$ of the considered plate; see Figure 1: (**a**) when ${\hat{n}}_{tr}\in \left[14.29,\;142.86\right]$, corresponding to $d_{c}\in \left[1.4\times 10^{-4},1.4\times 10^{-3}\right]$ or $d_{c}/d_{t}\in \left[0.2,\;2\right]$; and (**b**) when $d_{c}/d_{t}\in \left[0.02,2\right]$, corresponding to $d_{c}\in \left[1.4\times 10^{-5},1.4\times 10^{-3}\right]$ or ${\hat{n}}_{tr}\in \left[14.286,\;1428.571\right]$.

Figure 12 shows the dependencies of the bounds of the cross-sectional area of the laser-processed metal, $A_{l,inf}$ and $A_{l,sup}$, as well as their ratios $A_{l}/\left(1/2b_{l}b_{t}\right)$, where $A_{l}\in \left\{A_{l,inf},A_{l,sup}\right\}$ on the estimated number of the laser-processed tracks, ${\hat{n}}_{tr}$, calculated by Equations (6) or (10), and on the ratio $d_{c}/d_{t}$. The areas $A_{l,inf}$ and $A_{l,sup}$ were calculated by Equations (4), (5), (7)–(10) of the plate, shown in Figure 1, when the width of the laser-processed track $d_{t}=0.7$ mm and the width of the laser-processed area of the plate under consideration, $b_{l}=0.02$ m. The vertical dotted lines correspond to the minimum and maximum of ${\hat{n}}_{tr}$ or $d_{c}/d_{t}$ and laser-processing cases: case II when $d_{c}/d_{t}=2/3$; case III when $d_{c}/d_{t}=1.5$; and case IV when $d_{c}/d_{t}=2$; see Table 5.

Please note: in the present calculations, the numbers of tracks, $n_{tr}$, for cases II–IV were evaluated by Equations (6) or (10), i.e., $n_{tr}$ was not taken from Table 5.

As can be seen from Figure 12, $A_{l,inf}$ approaches $A_{l,sup}$ with increasing numbers of laser-processed tracks, ${\hat{n}}_{tr}$, or with a decrease in the ratio, $d_{c}/d_{t}$ or $d_{c}$. Also from Figure 12, it can be seen that the rate of increase of the areas, $A_{l}\in \left\{A_{l,inf},\;A_{l,sup}\right\}$, is practically constant with respect to increasing $n_{tr}$ within the interval $n_{tr}\in \left[\min\left\{n_{tr}\right\},\;28,57\right]$, corresponding to the interval, $d_{c}/d_{t}\in \left[1,\;max\{d_{c}\backslash d_{t}\}\right]$, or when $d_{c}/d_{t}\geq 1$.

More remarkably, the rate of increase of $A_{l}\in \left\{A_{l,inf},\;A_{l,sup}\right\}$ even increases with respect to decreasing $d_{c}/d_{t}$ within the interval $d_{c}/d_{t}\in [max\{d_{c}\backslash d_{t}\},\;1]$. However, as shown in Figure 12, the increase in the areas $A_{l}\in \left\{A_{l,inf},\;A_{l,sup}\right\}$ becomes slower with increasing $n_{tr}$ or decreasing $d_{c}/d_{t}$ or $d_{c}$ at $d_{c}/d_{t}<1$. 

Finally, the increase of $A_{l}\in \left\{A_{l,inf},\;A_{l,sup}\right\}$ practically does not change at a very large ${\hat{n}}_{tr}$. It should be noted that the biggest difference $max\left\{A_{l,sup}-A_{l,inf}\right\}=0.1924{\;\mathrm{mm}}^{2}$ and the ($max\left\{\left(A_{l,sup}-A_{l,inf}\right)/A_{l,sup}\right\}\cdot 100=6.66\%$) occurs at the largest value of ratio $d_{c}/d_{t}=2$, or at the smallest ${\hat{n}}_{tr}=14.29$.

**Figure 13:**
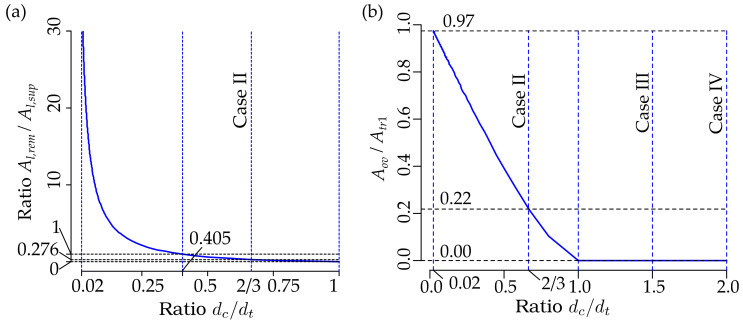
The dependencies of the ratios $A_{l,rem}/A_{l,sup}$ and $A_{ov}/A_{tr,1}$ on the ratio $d_{c}/d_{t}$ of the considered plate, see Figure 1: (**a**) $A_{l,rem}/A_{l,sup}$ versus $d_{c}/d_{t}\in \left[0.02,\;1\right]$; and (**b**) $A_{ov}/A_{tr,1}$ versus $d_{c}/d_{t}\in \left[0.02,\;2\right]$.

Figure 13 shows the dependency of the ratio of the area of the remelted metal to the supremum bound of the area of the laser-processed metal $A_{l,rem}/A_{l,sup}$ (Figure 13a), and the dependency of the ratio of the overlapping area on the cross-sectional area of the single laser-processed track $A_{ov}/A_{tr,1}$ on the ratio $d_{c}/d_{t}$ of the plate under consideration; see Figure 1, where the total area of the remelted metal is calculated as follows: $A_{l,rem}=(n_{tr}-1)A_{ov}=(b_{l}/d_{c})A_{ov}$. 

It is clear that $A_{l,rem}=A_{ov}=0$ as $d_{c}>d_{t}$ or $d_{c}/d_{t}>1$. As we can see from Figure 13a,b, the rate of the increase of the ratio $A_{ov}/A_{tr,1}$ on $d_{c}/d_{t}$ jumps sharply at $d_{c}/d_{t}=1$, and this rate is almost constant within the interval $d_{c}/d_{t}\in \left[0.02,\;1\right]$, since $A_{ov}/A_{tr,1}$ on $d_{c}/d_{t}$ depends very similarly to the line. However, the dependence of the ratio of the total area of the remelted metal to the supremum bound of the laser-processed metal $A_{l,rem}/A_{l,sup}$ increases very slowly at $d_{c}/d_{t}=1$, but becomes very steep with a higher ratio of $d_{c}/d_{t}$. 

The behavior of $A_{l,rem}/A_{l,sup}$ with respect to $d_{c}/d_{t}$ differs from $A_{ov}/A_{tr,1}$ due to the influence of the number of laser-processed tracks, $n_{tr}$. At relatively large $d_{c}/d_{t}$, the number $n_{tr}$ is small. Therefore, the contribution of $A_{ov}$ to $A_{l,rem}$ is also small. With decreasing $d_{c}/d_{t}$ the number $n_{tr}$ increases and, hence the contribution of increased $A_{ov}$ to $A_{l,rem}$ increases also.

As we can see from Figure 13a, the ratio $A_{l,rem}/A_{l,sup}=1$ as $d_{c}/d_{t}=0.405$. Therefore $A_{l,rem}>A_{l,sup}$ as $d_{c}/d_{t}<0.405$, and the difference $A_{l,rem}-A_{l,sup}$ or the ratio $A_{l,rem}/A_{l,sup}$ increases very quickly with increasing $d_{c}/d_{t}\geq 1$. It should be noted that the cross-sectional area of the remelted metal, $A_{l,rem}$ can be many times, ten- or even twenty-times, bigger than the supremum bound of the cross-sectional area of the laser-processed metal $A_{l,sup}$ at small values of $d_{c}/d_{t}$. Throughout this subchapter, a decrease in $d_{c}/d_{t}$ corresponds to increasing the number of laser-processed tracks, $n_{tr}$ and vice versa, while increasing $d_{c}/d_{t}$ corresponds to a decrease in $n_{tr}$.

The analysis above allows us to conclude that making a laser-processed layer is very inefficient at small values of $d_{c}/d_{t}$ or large values of $n_{tr}$, since a very large volume of the metal is melted repeatedly. For practical applications, it is reasonable to take the ratio, $d_{c}/d_{t}=1$. In this case, as we can see from Figure 12b, $A_{l}/\left(1/2b_{l}b_{t}\right)=0.785$. This means that the increase in the number of laser-processed tracks $n_{tr}$ or decrease of the distance between the tracks, $d_{c}$ or the ratio $d_{c}/d_{t}$ can increase the cross-sectional area of the laser-processed metal only up to $\left(1-0.785\right)\cdot 100=21.5\%$. However, as shown above, the cross-section of the remelted metal, or the ratio $A_{l,rem}/A_{l,sup}$, increases very quickly with decreasing $d_{c}$ or increasing $n_{tr}$. Hence, the efficiency of laser processing decreases very quickly. 

For practical applications, the optimal limit of ratio $d_{c}/d_{t}$ can be 0.405, since at this point $A_{l,rem}/A_{l,sup}=1$ and ratios $A_{l.inf}/\left(1/2b_{l}b_{t}\right)\approx 0.96$ and $A_{l.sup}/\left(1/2b_{l}b_{t}\right)\approx 0.97$. Therefore, a further increase in $n_{tr}$ or decrease in $d_{c}/d_{t}$ can increase the ratios $A_{l}/\left(1/2b_{l}b_{t}\right)$ only up to 4%. To increase the cross-sectional area of the laser-processed metal, $A_{l}$, it is better to increase the width, and hence the depth, of the laser-processed track $d_{t}$ than to increase $n_{tr}$ or decrease $d_{c}/d_{t}$.

The considerations given above concerning the ratios $A_{l}/\left(1/2b_{l}d_{t}\right)$, $A_{l,inf}/\left(1/2b_{l}d_{t}\right)$, $A_{l,sup}/\left(1/2b_{l}d_{t}\right)$, $A_{l,rem}/A_{l,sup}$, $A_{ov}/A_{tr,1}$ and the corresponding cross-section areas $A_{l,inf}$, $A_{l,sup}$, $\left(1/2b_{l}d_{t}\right)$, $A_{ov}$, $A_{tr,1}$, $A_{l,upp}=1/2b_{l}d_{t}$ are also valid for the corresponding ratios of the volumes $V_{l}/V_{l,upp}$, $V_{l,inf}/V_{l,upp}$, $V_{l,sup}/V_{l,upp}$, $V_{l,rem}/V_{l,sup}$, $V_{ov}/V_{tr,1}$ and the corresponding volumes $V_{l,inf}$, $V_{l,sup}$, $V_{ov}$, $V_{tr,1}$, $V_{l,upp}$ of the laser-processed metal, where V stands for the volume of the laser-processed metal and its indexes “l,inf”, “l,sup”, “l,upp”, $“V_{l,rem}”$, “ov”, and “tr,1” means the same as the corresponding indexes of the cross-sectional areas denoted by A. 

Since the above analysis, results, conclusions and recommendations are expressed in relative terms, then these considerations are also valid for other cases of plates, laser-processed areas, their widths $b_{l}$, the laser-processed track widths $d_{t}$, the distances between the track centers $d_{c}$, the number of laser tracks $n_{tr}$ and so on. 

#### 4.4.2. Axial Stiffness and Force—Strain Behavior of the Laser-Processed Plate

**Figure 14:**
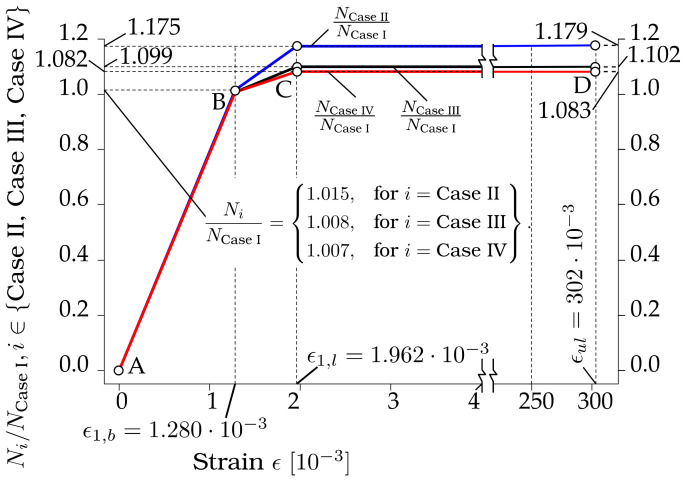
The dependencies of the ratios of the axial forces of the laser-processed plates, cases II, III and IV, to the unprocessed plate, case I: $N_{i}/N_{Case\;I}$, $i\in \left\{\mathrm{CaseI},\;\mathrm{Case}\;\mathrm{III},\;\mathrm{Case}\;\mathrm{IV}\right\}$ on strain $\epsilon \in \left[0,\epsilon_{ul}\right]=\left[0,\;302\cdot 10^{-3}\right]$ on strain *ϵ*.

The dependencies of the ratios of the axial forces of the laser-processed plates (see Figure 1, cases II, III and IV) to the unprocessed plate, case I: $N_{i}/N_{\mathrm{Case}\;I}$, $i\in \left\{\mathrm{CaseII},\;\mathrm{Case}\;\mathrm{III},\;\mathrm{Case}\;\mathrm{IV}\right\}$ on strain $\epsilon \in \left[0,\epsilon_{ul}\right]=\left[0,\;302\cdot 10^{-3}\right]$ are shown in Figure 14. The axial forces $N_{i}$, $i\in \left\{\mathrm{Case}\;I,\;\ldots ,\;\mathrm{Case}\;\mathrm{IV}\right\}$ were calculated according to Equations (1) and (2), using the properties of the base and laser-processed metals given in Table 7. The cross-sectional areas of the laser-processed metal of plate $A_{l}$ were calculated by the proposed Equations, (3), (7) and (11) when the number of the laser-processed sides $n_{s}=2$ and the number of the laser-processed tracks of one side of the plate, corresponding to cases I, II, III and IV $n_{tr}\in \left\{0,\;14,\;17,\;38\right\}$ is given in Table 5. The following cross-sectional areas were obtained: $A_{l}=0$ for case I, $A_{l}=1.1504\times 10^{-5}$ m^2^ for case II, $A_{l}=6.5423\times 10^{-6}$ m^2^ for case III, $A_{l}=5.3878\cdot 10^{-6}$ m^2^ for case IV. The cross-sectional areas of the base metal of the plate corresponding to cases I–IV, $A_{b}=A_{pl}-A_{l}$, where $A_{pl}=b_{pl}=4\times 10^{-5}$ m^2^ is the cross-sectional area of the plate; see Figure 1b. 

As we can see from Figure 14, the influence of laser processing is almost infinitesimal when $\epsilon \in \left[0,\;\epsilon_{1,b}\right]=\left[0,\;1.28\times 10^{-3}\right]$; see section AB in Figure 14. The maximum ratio, $\max\left\{N_{i}/N_{Case\;I},\;i\in \left\{\mathrm{CaseII},\;\mathrm{Case}\;\mathrm{III},\;\mathrm{Case}\;\mathrm{IV}\right\},\;\epsilon \in \left[0,\;\epsilon_{1,b}\right]\right\}=1.0015$. If the axial stiffness of the cross-section of the plate is B=N/ϵ, then we can say that laser processing does not have any influence on the axial stiffness of the plate when $\epsilon \in \left[0,\;\epsilon_{1,b}\right]$. However, the influence of laser processing on the ratio of the axial forces $N_{i}/N_{Case\;I}$ increases with increasing strain within the interval $\epsilon \in \left[\epsilon_{1,b},\;\epsilon_{1,l}\right]=\left[1.28\times 10^{-3},1.962\times 10^{-3}\right]$ and remains constant when $\epsilon \in \left[\epsilon_{1,l},\;\epsilon_{ul}\right]=\left[1.962\times 10^{-3},302\times 10^{-3}\right]$, where hereafter $\epsilon_{ul}=\epsilon_{ul,b}=\epsilon_{ul,l}=302\times 10^{-3}$; see sections BC and CD in Figure 13.

As shown in Figure 14, laser processing increases the axial forces $N_{i}$ and hence the axial stiffness $B_{i}$, $i\in \left\{\mathrm{Case}\;\mathrm{II},\;\mathrm{Case}\;\mathrm{III},\;\mathrm{Case}\;\mathrm{IV}\right\}$, about 17.5% for case II, 9.9% for case III, and 8.2% for case IV. For practical structural calculations, it is reasonable to assume that the plate’s bearing capacity corresponds to the axial force $N_{i}$ at $\epsilon =\epsilon_{1,l}$, since the increase of the ratio $N_{i}/N_{Case\;I}$ within the interval $\left[\epsilon_{1,l},\;\epsilon_{ul}\right]$ is very low: $\left(1.179–1.175\right)=2.5\%$ for case II; 1.102−1.009=0.9% for case III; and 1.083−1.082=0.1% for case IV. 

**Figure 15:**
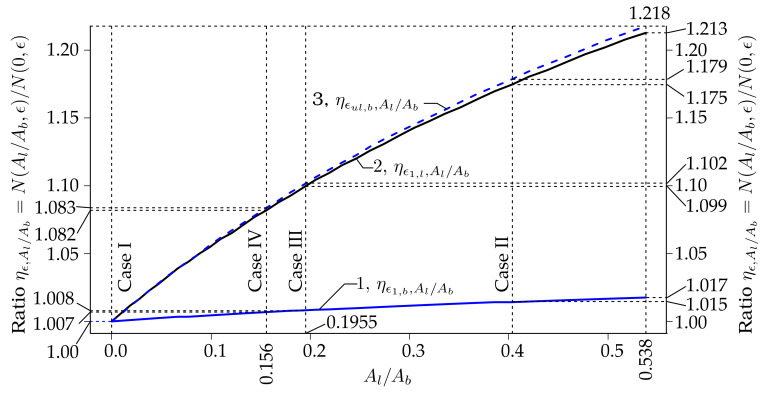
The dependencies of the ratios of the axial forces $\eta_{\epsilon ,A_{l}/A_{b}}=N\left(A_{l}/A_{b},\epsilon \right)/N\left(0,\epsilon \right),$ at $\epsilon \in \left\{\epsilon_{1,b},\epsilon_{1,l},\epsilon_{ul}\right\}$ with respect to the ratio of the cross-section areas of the laser-processed and base metals $A_{l}/A_{b}\in \left[0,\;0.538\right]$ of the plate shown in Figure 1.

The dependencies of the ratios of the axial forces $\eta_{\epsilon ,A_{l}/A_{b}}=N\left(A_{l}/A_{b},\epsilon \right)/N\left(0,\epsilon \right),$ at $\epsilon \in \left\{\epsilon_{1,b},\epsilon_{1,l},\epsilon_{ul}\right\}$ with respect to the ratio of the cross-sectional areas of the laser-processed and base metals $A_{l}/A_{b}\in \left[0,\;0.538\right]$ of the plate shown in Figure 1 are shown in Figure 15. In the situation when $A_{l}=0$, since $A_{l}/A_{b}=0$ also corresponds to the laser-unprocessed plate, i.e., case I, then $N\left(0,\epsilon \right)=N_{\mathrm{Case}\;I}$. In these calculations, it was assumed that the cross-sectional area of the laser-processed metal attains values from the interval: $A_{l}\in \left[0,\;A_{l,up}\right]=\left[0,\;2\times 1/2b_{l}d_{t}\right]=\left[0,\;1.4\times 10^{-5}\right]$. Since $A_{b}=A_{pl}-A_{l}$, where $A_{pl}=b_{pl}\times 2\times 10^{-3}=4\times 10^{-5}$, then $max\left\{A_{l}/A_{b}\right\}=1.4/\left(4-1.4\right)=0.5385$. Thus, $max\left\{A_{l}/A_{b}\right\}=0.5385$ corresponds to the upper bound of the cross-sectional area of the laser-processed metal when $A_{l,up}=n_{s}1/2b_{l}d_{t}=20\times 10^{-3}\times 0.7\times 10^{-3}=1.4\times 10^{-3}$ of the two-side laser-processed plate; see Figure 1b. Other parameters used to draw Figure 15 were the same as in Figure 14; see the explanations given at the beginning of the present subchapter.

As we can see from Figure 15, the ratios $\eta_{\epsilon ,A_{l}/A_{b}}$ on $A_{l}/A_{b}$ do not change according to the linear law, as in the case of the dependence of the N on $A_{l}$ when $A_{b}$ is constant, according to Equation (2). This happens since the cross-sectional area of the base metal $A_{b}=A_{pl}-A_{l}$, i.e., $A_{b}$ also changes when $A_{l}$ changes. Also, it is clearly visible that the ratio $A_{l}/A_{b}$ affects the ratios $\eta_{\epsilon ,A_{l}/A_{b}}$, especially when $\epsilon \in \left[\epsilon_{1,b},\;\epsilon_{1,l}\right].$ Thus, when $\epsilon \in \left\{0,\epsilon_{1,b}\right\}$, then the relative difference $\Delta_{max}\eta_{\epsilon ,A_{l}/A_{b}}=max\left\{\eta_{\epsilon ,A_{l}/A_{b}}-\eta_{\epsilon ,0},A_{l}/A_{b}\in \left\{0,\;0.538\right\}\right\}=\eta_{\epsilon ,0.538}-\eta_{\epsilon ,0}=1.7\%$, while when $\epsilon \in \left\{\epsilon_{1,b},\epsilon_{1,l}\right\}$, then $\Delta_{max}\eta_{\epsilon ,A_{l}/A_{b}}=21.3\%$; and when $\epsilon \in \left\{\epsilon_{1,b},\epsilon_{ul}\right\}$, then $\Delta_{max}\eta_{\epsilon ,A_{l}/A_{b}}=21.8\%$. When the strain increases from $\epsilon_{1,l}$ to $\epsilon_{ul}$ and $A_{l}/A_{b}=0.538$, then the relative difference $\Delta_{max}\eta_{\epsilon ,A_{l}/A_{b}}=\eta_{\epsilon_{ul},0.538}-\eta_{\epsilon_{l},0.538}=0.5\%$. From Figure 15, we can see that the biggest possible increase of the axial force is when $A_{l}/A_{b}=A_{l,sup}/\left(A_{pl}-A_{l,sup}\right)=0.538$ is $\eta_{\epsilon_{ul},0.538}-\eta_{\epsilon_{l},0.538}=1.213-1.017=19.6\%$.

## 5. Conclusions

1. According to the results of these experiments and computer simulations of elastoplastic deformation, it is established that the local laser processing can be used to increase the flexural stiffness of structural elements made from thin-sheet steel (1.0402) up to 5% and to decrease its deformation or deflection under similar workload up to 13–24%, instead of applying complex geometric shapes, additional stiffening elements or heat treatment.
2. The steel hardness and strength in the processed zones increased by up to 50% compared to untreated areas, which led to an increase in the flexural rigidity of the laser-processed metal plate and the maximum level of endured load without plastic deformation.
3. It has been established that the stiffness of thin-sheet steel depends on and can be altered by the parameters of laser processing, the area treated, the number of laser tracks, and the distance between tracks.
4. The conducted analytical modeling showed that, when the ratio of the distance between the centers of the laser-processed tracks and their width equals 1, i.e., no gaps exist between the laser-processed tracks, then the volume ratio of the laser-processed metal to its greatest possible volume is 0.785.
5. For practical applications, the smallest reasonable ratio of the distance between the centers of the laser-processed tracks to their width is 0.405. In this case, the volume of the repeatedly melted metal equals the volume of laser-processed metal; and the ratio of the volume of laser-processed metal to its greatest possible volume is 0.97.
6. To produce a greater volume of laser-processed metal, we suggest increasing the width of the laser-processed metal track by increasing the laser power, instead of decreasing the distance between the laser-processed tracks centers below 0.405 of their width.
7. The analytical modelling of the axial force-strain behavior of the laser-processed plates showed that the axial stiffness of the plate cross-section increases up to 21.3% at the strain corresponding to the yield point of the laser-processed metal when the distance between the laser-processed tracks equals 2/3 of their width.

## Figures and Tables

**Table 1:** Chemical composition of samples from steel (1.0402).

Chemical Elements [wt.%]					
C	Si	Mn	P	S	Cr + Mo + Ni
0.18	0.18	0.45	0.02	0.02	0.15

**Table 2:** Mechanical properties of samples from steel (1.0402).

Elastic Modulus *E*, [GPa]	Yield Strength $\sigma_{0.2},\;[\mathrm{MPa}]$	Tensile Strength $\sigma_{B}\;[\mathrm{MPa}]$	Relative Extension *A*, [%]	Hardness, [HV]
200	256	410	30	125

**Table 3:** Physical properties of low-carbon steel (1.0402) [16].

Melting Temperature, *T*_m_, [K]	Ambient Temperature, *T*_0_, [K]	Absorptivity, *A*	Reflection Coefficient, *R*	Density, *ρ*, [kg/m^3^]	Specific Heat, *c*, [J/kg·K]
1733	295	0.09	0.91	7800	462

**Table 4:** Parameters of local laser treatment.

Depth *h*, [mm]	Speed *v*, [mm/min]	Frequency *f*, [Hz]	Single Pulse Energy *E*_p_, [J]	Spot Size *d*, [mm]	Peak Power *P*_p_, [kW]	Overlap Coefficient *P*_er_, [%]	Critical Power *P*_d1_, [W/cm^2^]
0.346	240	10	14.4	3	2.8	98.6	4.11∙10^5^

**Table 5:** Force–deflection experiment cases.

Experiment Case Notation	Laser Processing Case and Number of One-Side Laser Tracks	Loading Variant	Position and Stress-Strain State of the Laser-Processed Layer
Case I	Case I, 0 tracks		there are no laser-processed layers
Case II A		A	at the top under compression
Case II B	Case II, 38 tracks	B	at the bottom under tension
Case II C		C	at the top under compression, and at the bottom under tension
Case III A		A	at the top under compression
Case III B	Case III, 17 tracks	B	at the bottom under tension
Case III C		C	at the top under compression, and at the bottom under tension
Case IV A		A	at the top under compression
Case IV B	Case IV, 14 tracks	B	at the bottom under tension
Case IV C		C	at the top under compression, and at the bottom under tension

**Table 6:** The laser processing and variants of geometry of the laser-processed area and bending loading.

Laser Processing Case	Loading Variants	Number of the Laser-Processing Tracks [pc]	Volume of the Laser-Processed Metal [mm^3^]	Ratios of Volumes of the Laser-Processed Metal to the Total Bended Volume [%]
Case I		0	0	0
Case II	A	38	277.3	9.9
	B	38	277.3	9.9
	C	76	554.6	19.8
Case III	A	17	261.5	9.3
	B	17	261.5	9.3
	C	34	523	18.6
Case IV	A	14	215.4	7.7
	B	14	215.4	7.7
	C	28	430.8	15.4

**Table 7:** Parameters of base metal and laser-processed layer used to simulate elastoplastic deformation of samples [24,25].

Material	Modulus of Elasticity, *E* [GPa]	Shear Modulus, *G* [GPa]	Yield Strength, $\sigma_{0.2}\;[\mathrm{MPa}]$	Ultimate Strength, $\sigma_{B}\;[\mathrm{MPa}]$	Poisson’s Ratio *ν*	Strength Coefficient $E_{1}\;[\mathrm{GPa}]$	Yield Stress Strains $\epsilon_{1}$ $\left(10^{-3}\right)$	Ultimate Strains $\epsilon_{ul}$ ($10^{-3}$)
Base metal	200	78.1	256	410	0.28	0.512	1.280	302.0
Laser-processed layer	210	82	412	665	0.28	0.843	1.962	302.0

**Table 8:** Chemical compositions (by energy-dispersive X-ray spectroscopy analysis) of surface regions.

Region	Chemical Elements [wt.%]				
	C	Fe	Si	Mn	Other
Laser-processed area	1.49	97.28	0.20	0.42	0.61
Basic metal	1.13	97.67	0.21	0.43	0.56

**Table 9:** The maximum Von Mises equivalent stresses (in MPa), and corresponding forces $F_{calc}$ (in N) of the finite element analysis (FEA) simulation of the bending of the differently laser-processed plates at different imposed vertical displacements $w\in \left\{0.5,1.0,1.5,2.0\right\}$ mm of the middle point B.

Imposed Vertical Displacement of the Point B, see Figure 2b, [mm]	0.5	1.0	1.5	2.0
**Laser-Unprocessed Plate (Case I)**				
Maximum von Mises stress, [MPa]	176	293	395	409
Bending force $F_{calc}$, [N]	179	231	269	290
**Laser-Processed Plate (Case IIA)**				
Maximum von Mises stress, [MPa]	217	369	460	468
Bending force $F_{calc}$, [N]	215	318	390	409
**Laser-Processed Plate (Case IIB)**				
Maximum von Mises stress, [MPa]	219	372	463	470
Bending force $F_{calc}$, [N]	217	320	392	410
**Laser-Processed Plate (Case IIC)**				
Maximum von Mises stress, [MPa]	228	385	497	510
Bending force $F_{calc}$, [N]	228	345	417	442
**Laser-Processed Plate (Case IIIA)**				
Maximum von Mises stress, [MPa]	209	350	431	460
Bending force, [N]	205	311	375	394
**Laser-Processed Plate (Case IIIB)**				
Maximum von Mises stress, [MPa]	210	352	432	462
Bending force $F_{calc}$, [N]	206	312	378	398
**Laser-Processed Plate (Case IIIC)**				
Maximum von Mises stress, [MPa]	223	382	485	505
Bending force $F_{calc}$, [N]	222	334	411	428
**Laser-Processed Plate (Case IVA)**				
Maximum von Mises stress, MPa	199	332	425	448
Bending force $F_{calc}$, N	184	298	354	378
**Laser-Processed Plate (Case IVB)**				
Maximum von Mises stress, MPa	200	338	426	452
Bending force $F_{calc}$, N	187	300	356	381
**Laser-Processed Plate (Case IVC)**				
Maximum von Mises stress, MPa	220	380	480	502
Bending force $F_{calc}$, N	219	328	403	419

**Table 10:** Experimental and modeled bending forces $F_{exp}$ and $F_{calc}$ respectively, and their relative differences $d_{F,r}$ at different deflections w.

Bending Forces $F_{exp}$, $F_{calc}$ and Relative Differences $d_{F,r}=\left(F_{exp}-F_{calc}\right)/F_{calc}$	Case I	Case IIA	Case IIB	Case IIC	Case IIIA	Case IIIB	Case IIIC	Case IVA	Case IVB	Case IVC
Deflection, 0.5 mm										
$F_{exp}$, [N]	200	226	228	242	220	221	236	209	211	230
$F_{calc}$, [N]	179	215	217	228	205	206	222	184	187	219
$d_{F,r}$, [%]	12	5	5	6	7	7	6	13	13	5
Deflection, 1 mm										
$F_{exp}$, [N]	261	365	368	387	352	356	376	333	336	370
$F_{calc}$, [N]	231	318	320	345	311	312	334	298	300	328
$d_{F,r}$, [%]	13	15	15	13	13	14	13	12	12	13
Deflection, 1.5 mm										
$F_{exp}$, [N]	282	425	426	455	412	413	437	402	403	431
$F_{calc}$, [N]	269	390	392	417	375	378	411	354	356	403
$d_{F,r}$, [%]	5	9	9	9	10	9	6	13	13	7
Deflection, 2 mm										
$F_{exp}$, [N]	296	445	447	475	439	440	463	421	423	455
$F_{calc}$, [N]	290	409	410	442	394	398	428	378	381	419
$d_{F,r}$, [%]	2	9	9	7	11	10	8	11	11	9

## Nomenclature

$A_{l}$ and $A_{b}$	The total areas of the cross-sections of the laser-processed and unprocessed metals respectively
$A_{l,sup}$ and $A_{l,inf}$	The supremum and infimum bounds of the cross-section area of the laser-processed metal
$A_{pl}$	The cross-sections area of the plate
$A_{l,rem}$	The cross-sections area of the remelted laser-processed metal due to overlapping the adjacent laser-processed metal tracks
$A_{ov}$	The cross-sections area of the two adjacent overlapping laser-processed metal tracks
$A_{tr,1}$	The cross-sections area of the single track of the laser-processed metal
B	Cross-section axial stiffness of a plate
$b_{pl}$	Width of a plate
$b_{l}$	Width of the laser-processed area
$d_{t}$ and $d_{c}$	The width of the laser-processed tracks and the distance between centers of two adjacent laser-processed tracks respectively
$E_{i}$, $E_{1,i}$, $G_{i}$, and $\nu_{i}$, $i\;\in \left\{b,l\right\}$	Modulus of elasticity, strength coefficient, shear modulus and Poisson’s ratio of the base metal, when i=b, and the laser-processed metal, when i=l respectively
FEA	Finite element analysis
$F_{calc}$	The vertical force imposed in the middle of the plate span calculated by FEA
$F_{exp}$	Experimentally determined vertical force imposed in the middle of the plate span
N, $N_{b}$ and $N_{l}$	The axial forces acting in the total cross-section of the plate, in the cross-section of the base metal and in the cross-section of the laser-processed metal respectively
$n_{s}\in \left\{1,2\right\}$	The number of laser-processed sides
$n_{tr}$ and ${\hat{n}}_{tr}$	Number and estimated number of the laser-processed tracks
w	The deflection or the vertical displacement of the plate midpoint
ϵ	Linear strain
$\epsilon_{1,i}$, $i\;\in \left\{b,l\right\}$	Yield stress strains of the base, i.e., laser-unprocessed, and laser-processed metals respectively
σ	The normal stress
$\epsilon_{ul}$, $\epsilon_{ul,i}$, $i\in \left\{b,l\right\}$	Ultimate strains of the base, i.e., laser-unprocessed, and laser-processed metals respectively
$\sigma_{b}$ and ${\hat{\sigma }}_{b}$	The stress-strain function and its approximation of the base, i.e., laser-unprocessed, metal respectively
${\hat{\sigma }}_{l}$	The approximation of the stress-strain function of the laser-processed metal
$\sigma_{0.2,i}$, $\sigma_{B,i}$, $i\in \left\{b,l\right\}$	Yield strengths and ultimate strength of the base metal and the laser-processed metal respectively
